# Gaps in Routine Childhood Immunization Among Low-Income Children at 24 Months (2019–2024)

**DOI:** 10.1016/j.focus.2026.100510

**Published:** 2026-05-08

**Authors:** Kurt C. Stange, Rose Goueth, Anna R. Templeton, Matthew W.H. Jones, Claudia Der-Martirosian, Suparna M. Navale, Rae Crist, Nicole Cook

**Affiliations:** 1 Center for Community Health Integration, Case Western Reserve University, Cleveland, Ohio; 2 Research Department, OCHIN, Inc., Portland, Oregon

**Keywords:** Childhood immunizations, community health centers, low-income, public health

## Abstract

**Introduction:**

Vaccination rates declined during the COVID-19 pandemic and have not fully rebounded after pandemic. Declines in routine vaccination rates can lead to the increased emergence or reemergence of childhood diseases that are currently not widely circulating in the U.S. This study assessed the rates of required childhood vaccinations at 24 months from 2019 to 2024 by patient demographics and utilization.

**Methods:**

Electronic health record data from 213 community-based primary care health centers serving underserved and low-income populations in 10 states were used to calculate the routine vaccination metric over 6 years. Up-to-date rates were calculated for children with and without well-child visits. Rates were also calculated with and without the inclusion of the influenza vaccine.

**Results:**

Up-to-date vaccination rates at 24 months increased from 2019 to 2020 and declined from 2021 to 2024. The percentage of children who were up to date with all vaccines ranged from 42% in 2020 to a low of 30% in 2024. Rates were higher when the influenza vaccine was excluded and among children who had a well-child visit.

**Conclusions:**

Routine childhood vaccination rates at 24 months declined during the COVID-19 public health emergency and have continued to decline since then. Targeted strategies are needed to increase routine childhood vaccination coverage.

## HIGHLIGHTS

• Declines in routine childhood vaccinations during the pandemic persist.
• Children from higher-income families are more likely to be up to date.
• Collaboration between providers and state and national initiatives is needed.

## INTRODUCTION

Adherence to the recommended routine childhood vaccination schedule has shown little to no improvement over the past decade.^1^ Disruptions in health care during the coronavirus disease 2019 (COVID-19) public health emergency (PHE) exacerbated the challenges to completing routine vaccinations for children reaching the age of 24 months, particularly among low-resource groups and individuals facing persistent barriers to care.^2^^,^^3^

Community-based health centers, which serve as the nation’s primary care safety net, are frontline providers of vaccines to children.^4^, ^5^, ^6^ Using data from 213 health center organizations sharing a common electronic health record (EHR) system, this study evaluated the proportion of children who were up to date with routine childhood vaccinations at the age of 24 months from the prepandemic year 2019 through 2024.

## METHODS

### Study Sample

This study used EHR data from the Accelerating Data Value Across a National Community Health Center Network Clinical Research Network, a member of PCORnet. Accelerating Data Value Across a National Community Health Center Network partners serve Federally Qualified Health Centers, public health departments, and community-based health centers that deliver care to people who are often low income and publicly insured or uninsured. The study sample is limited to primary care clinics from OCHIN’s (not an acronym) multisite network of community-based health centers.^4^^,^^5^

Included are clinics that had at least 200 eligible children in any study year and that are located in states with enabled bidirectional state immunization registries. Receipt of immunizations includes vaccines administered in clinics and those administered outside clinics, updated manually in the EHR or through interoperable state vaccine registries.

### Measures

To determine up-to-date status, the Uniform Data System childhood immunization metric, which aligns with the CMS117v10 electronic clinical quality measure specification, was used. The 2022 metric was selected because this was the baseline year for trended data analysis.^6^ The 2022 measure includes the following: percentage of children who turned age 2 years in the past year and have evidence in their EHR of receiving 4 diphtheria, tetanus, and acellular pertussis (DTaP); 3 polio (inactivated poliovirus vaccine); 1 measles, mumps, and rubella; 3 or 4 *Haemophilus influenzae* type B; 3 hepatitis B; 1 chickenpox (varicella-zoster virus); 4 pneumococcal conjugate (PCV); 1 hepatitis A; 2 or 3 rotavirus; and 2 influenza vaccines by their second birthday. Evidence of an adverse reaction or seropositivity to a vaccine in the patient EHR satisfied the requirement for evidence of receipt of the recommended vaccine. All children who turned age 2 years and had an initial or established visit at the health center during the year were included in the denominator. Patients receiving hospice care were excluded from the denominator. Vaccine administration codes were updated in 2023 and 2024 to ensure that new vaccines were included in the calculations.

Because the influenza vaccine is administered each year during specific months, there is no catch-up option for any child who misses the influenza vaccine in 1 year to ever be up to date. For this reason, annual up-to-date rates from 2019 to 2024 were plotted with and without the influenza vaccine.

### Statistical Analysis

Subgroup trends were evaluated by race/ethnicity, federal poverty level (FPL), well-child visit, and clinic utilization (1, 2, or >2 visits in the year). It was anticipated that children with a well-child visit would be more likely to have completed routine vaccinations. Therefore, trended rates were compared with and without a well-child visit during the measurement year. This study was reviewed by Case Western Reserve University’s IRB.

## RESULTS

The number of children who turned age 24 months during a study year ranged from 20,733 to 18,175. Overall, most patients in the study were Hispanic and were part of a family with FPL ≤138%. The percentage of patients with a well-child visit fell from 83.1% in 2019 to 77.1% in 2020 and then increased slightly in the ensuing years. Clinics with patients included in the study were most likely to be in California, Oregon, and Massachusetts (Table 1).

**Table 1:** Demographic Characteristics of Children Aged 24 Months as of December 31 in 213 Primary Care Health Center Clinics (2019–2024)

	2019		2020		2021		2022		2023		2024	
Characteristics	*n*	%	*n*	%	*n*	%	*n*	%	*n*	%	*n*	%
Total patients	20,733		18,382		18,311		18,461		18,175		18,526	
Race/ethnicity												
Asian	1,037	5.0%	969	5.3%	915	5.0%	1,085	5.9%	1,118	6.2%	1,137	6.1%
Black, non-Hispanic	4,044	19.5%	3,513	19.1%	3,534	19.3%	3,506	19.0%	3,162	17.4%	2,975	16.1%
Hispanic	8,962	43.2%	7,996	43.5%	7,919	43.2%	7,763	42.1%	7,594	41.8%	7,950	42.9%
Other multiethnic	384	1.9%	378	2.1%	369	2.0%	315	1.7%	350	1.9%	364	2.0%
White, non-Hispanic	4,816	23.2%	4,034	21.9%	3,905	21.3%	3,688	20.0%	3,485	19.2%	3,462	18.7%
Unknown	1,490	7.2%	1,492	8.1%	1,669	9.1%	2,104	11.4%	2,466	13.6%	2,638	14.2%
Federal poverty level												
≤138%	13,362	64.4%	11,524	62.7%	11,897	65.0%	12,356	66.9%	12,254	67.4%	12,620	68.1%
>138%	2,950	14.2%	2,311	12.6%	2,495	13.6%	2,706	14.7%	2,488	13.7%	2,358	12.7%
Unknown	4,421	21.3%	4,547	24.7%	3,919	21.4%	3,399	18.4%	3,433	18.9%	3,548	19.2%
Total visits in year		0.0%										
1	5,670	27.3%	6,119	33.3%	5,631	30.8%	5,403	29.3%	5,573	30.7%	5,507	29.7%
2	4,794	23.1%	4,575	24.9%	4,503	24.6%	4,322	23.4%	4,518	24.9%	4,364	23.6%
≥3	10,269	49.5%	7,688	41.8%	8,177	44.7%	8,736	47.3%	8,084	44.5%	8,655	46.7%
Clinic state												
California	7,336	35.4%	6,596	35.9%	6,518	35.6%	6,690	36.2%	6,613	36.4%	6,706	36.2%
Georgia	149	0.7%	381	2.1%	446	2.4%	409	2.2%	409	2.3%	385	2.1%
Indiana	1,032	5.0%	936	5.1%	975	5.3%	990	5.4%	926	5.1%	1,357	7.3%
Massachusetts	4,082	19.7%	3,505	19.1%	3,539	19.3%	3,606	19.5%	3,375	18.6%	2,939	15.9%
Minnesota	274	1.3%	275	1.5%	257	1.4%	283	1.5%	269	1.5%	271	1.5%
North Carolina	402	1.9%	380	2.1%	390	2.1%	459	2.5%	443	2.4%	457	2.5%
Ohio	1,908	9.2%	1,631	8.9%	1,638	8.9%	1,565	8.5%	1,527	8.4%	1,737	9.4%
Oregon	4,235	20.4%	3,549	19.3%	3,393	18.5%	3,281	17.8%	3,366	18.5%	3,454	18.6%
Washington	834	4.0%	659	3.6%	631	3.4%	667	3.6%	708	3.9%	620	3.3%
Wisconsin	481	2.3%	470	2.6%	524	2.9%	511	2.8%	539	3.0%	600	3.2%
Well-child visit												
Yes	17,234	83.1%	13,635	74.2%	14,453	78.9%	14,594	79.1%	14,303	78.7%	14,286	77.1%
No	3,499	16.9%	4,747	25.8%	3,858	21.1%	3,867	20.9%	3,872	21.3%	4,240	22.9%

Table 2 shows that the percentage of children up to date with routine vaccinations, less the influenza vaccine, declined for every subgroup, except for children who self-report as White, non-Hispanic. Children in families with FPL ≤138% saw a decline of 7.7% from 2019 to 2024 (60.9%–53.2%). Dose effects over the study timeline were seen between number of visits and up-to-date rates and between well-child visits and up-to-date rates. There was variation by state. Up-to-date vaccination rates increased in Georgia, Indiana, and North Carolina between 2019 and 2024, whereas in all other states, rates declined (Appendix Figure 1, available online).

**Table 2:** Percent of Children With Up-To-Date Routine Childhood Vaccinations (Birth Through 24 Months), Less Influenza, by Race/Ethnicity, Federal Poverty Level, Number of Patient Visits in Year, Well-Child Visits and Clinic State (2019–2024)

Characteristic	2019	2020	2021	2022	2023	2024	% point change 2019–2024
Total patients	20,733	18,382	18,311	18,461	18,175	18,526	
Race/ethnicity							
Asian	52.1%	53.1%	54.8%	42.9%	41.5%	41.7%	−10.4%
Black, non-Hispanic	55.8%	54.9%	51.8%	50.5%	50.7%	46.4%	−9.4%
Hispanic	70.0%	72.7%	68.8%	67.4%	66.6%	63.6%	−6.4%
Other multiethnic	53.9%	58.7%	53.4%	51.4%	46.3%	47.8%	−6.1%
White, non-Hispanic	52.4%	56.9%	57.3%	54.6%	52.1%	56.1%	3.6%
Unknown	56.9%	56.7%	54.8%	48.3%	48.0%	49.2%	−7.7%
Federal poverty level							
≤138%	60.9%	62.8%	60.2%	55.7%	54.5%	53.2%	−7.7%
>138%	67.5%	71.2%	68.1%	65.2%	64.4%	64.1%	−3.3%
Unknown	57.1%	60.3%	58.0%	59.3%	58.5%	59.1%	2.0%
Total visits in year							
1	45.6%	50.1%	45.6%	41.0%	42.6%	42.1%	−3.5%
2	56.9%	62.4%	59.0%	55.0%	54.7%	50.3%	−6.5%
≥3	71.5%	74.2%	72.3%	69.5%	67.3%	67.1%	−4.4%
Clinic state							
California	62.0%	66.8%	64.7%		60.8%	61.3%	
Georgia	54.4%	73.2%		76.8%	76.5%	74.0%	19.7%
Indiana	48.0%	48.0%	49.9%	51.3%	51.7%	58.7%	10.7%
Massachusetts	72.8%	72.5%	66.5%	65.0%	64.7%	60.8%	−12.0%
Minnesota	58.4%	48.7%	45.5%	46.6%	47.6%	46.1%	−12.3%
North Carolina	64.7%	65.8%	66.2%	65.8%	72.7%	72.2%	7.5%
Ohio	55.8%	65.2%	63.6%	60.1%	59.4%	52.7%	−3.1%
Oregon	57.2%	58.0%	55.7%	48.2%	44.2%	45.0%	−12.2%
Washington	36.1%	38.4%	37.9%	33.3%	34.5%	36.3%	0.2%
Wisconsin	43.5%	40.9%	38.2%	35.0%	37.5%	32.2%	−11.3%
Well-child visit							
Yes	66.0%	69.2%	67.0%	65.3%	64.4%	62.7%	−3.3%
No	36.2%	46.2%	37.4%	29.1%	27.8%	32.1%	−4.1%

**Figure 1:**
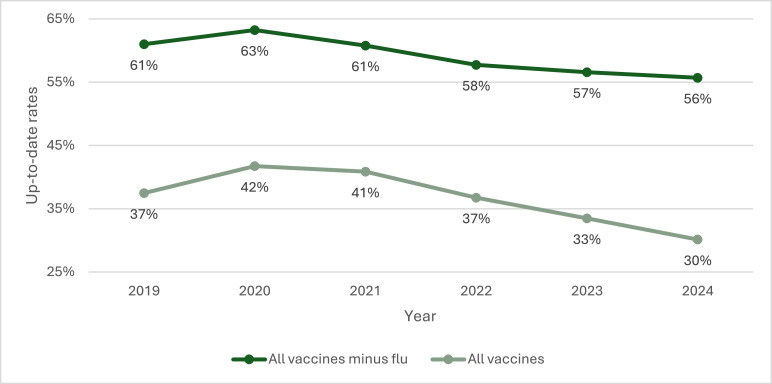
Up-to-date 24-month childhood vaccination rates.

The overall childhood vaccination rate increased slightly from 2019 to 2020 but declined in each subsequent year through 2024. This trend was seen both for the full set of routine vaccinations and for the set less the influenza vaccine (Figure 1).

A review of individual vaccines demonstrated that each vaccine declined from 2020 to 2023 and then, except for influenza, increased slightly in 2024 (Appendix Figure 2, available online). Influenza, DTaP, and PCV were less likely to be up to date than other vaccines. Among children with a well-child visit, most vaccines declined slightly between 2020 and 2021 and then remained steady between 2021 and 2024, except for rotavirus and DTaP, which saw some dips and peaks, and PCV and influenza, which declined steadily from 2020 to 2024 (Appendix Figure 3, available online).

## DISCUSSION

This study provides a comprehensive picture of routine childhood vaccinations (birth through the age of 24 months) from 2019 to 2024 among 213 clinics serving low-income and vulnerable populations in 10 states. Prior to the PHE, up-to-date vaccination rates were increasing in 2019 and 2020 but then declined in 2021. Although up-to-date rates improved from 2022 to 2024, they did not rebound to 2020 levels. This is consistent with national data that show that although vaccination coverage rebounded for most groups, children living in poverty and those in rural areas continue to experience lower rates of routine vaccination.^7^^,^^8^

Myriad reasons for lower vaccine rates include vaccine hesitancy, distance to receive a vaccination, negative prior experiences, fear of pain, vaccine literacy, and others.^9^, ^10^, ^11^, ^12^, ^13^, ^14^, ^15^, ^16^ The PHE added new challenges to routine vaccinations, particularly for lower-income groups.^2^ In addition to traditional reasons contributing to lower vaccine rates, receipt of vaccinations during PHE was challenged by a decline in in-person visits in clinics, the closure of school-located vaccination clinics, and changes in school exemption policies .^17^, ^18^, ^19^, ^20^, ^21^, ^22^, ^23^

Health centers provide front-line care to 1 out of 3 people living in poverty and continued to lead efforts to care for low-income communities throughout the pandemic.^24^ They are also key partners in childhood vaccinations; most participate in the Vaccines for Children program, which provides free vaccines for children who meet certain criteria, including having public or no insurance.^25^ Annual reporting for children up to date with routine vaccinations is also a core annual quality measure.^26^

### Limitations

This study has limitations. First, the study is limited to available data from clinics in 10 states and cannot be generalized nationally. Second, EHR data may be incomplete, resulting in underreporting of completed vaccinations. The study team confirmed that interoperable vaccine state registries are enabled for each state with data in the study. However, it is possible that patients receive care outside of the state and that such data were never entered either in the patient EHR or the state registry. It is also possible, though unlikely, that some vaccine codes were missing to identify all vaccines in EHRs. Third, the study team remains cautious about suggesting that the overall decline in routine vaccination rates be singularly attributed to disruptions in care or changes in exemption policies during the PHE. During the PHE, vaccine hesitancy increased.^8^ More importantly, the relative contribution of variables that may explain both the decline in coverage and the ongoing gaps in coverage between low-income and high-income populations is unknown. Similarly, the study team is unable to directly explain increases in vaccine completion from 2019 to 2020 in California, Georgia, and Ohio, when a drop in vaccination rates would be expected owing to pandemic-related closures in March 2020. The study team posits that this could partly be attributed to the administration of most childhood vaccinations by 12 months (e.g., 2019 for children who turned age 2 months in 2020). It could also partly be due to health centers allocating dedicated resources during the public health emergency to prioritize outreach and completion of immunization schedules. More nuanced review of data trends across states and deeper investigation of health center policies and practices during the pandemic are required to fully explain trends by subgroups and individual vaccines.

## CONCLUSIONS

Declines in routine vaccination rates can lead to the increased emergence or reemergence of childhood diseases that are currently not widely circulating in the U.S. Ongoing collaboration between providers and state and national initiatives is needed to ensure that low-income children receive routine vaccinations per national guidelines.

## Appendix Supplementary materials

Image, application 1

## Footnotes

Supplementary material associated with this article can be found in the online version at doi:10.1016/j.focus.2026.100510.
