# Supplementary material for: Gaps in Routine Childhood Immunization Among Low-Income Children at 24 Months (2019–2024)
Source: AJPM Focus. 2026 May 8;5(5):100510. doi: 10.1016/j.focus.2026.100510 (PMC13427420; doi:10.1016/j.focus.2026.100510)

Appendix

AJPM Focus

Gaps in routine childhood immunization among low-income children at 24 months (2019-2024)

Kurt C. Stange, MD, PhD[1], Rose Goueth, PhD, MS [2], Anna R. Templeton, DNP[2], Matthew W. H. Jones, MS[2], Claudia Der-Martirosian, PhD[2], Suparna Navale, PhD[2], Rae Crist, MS[2], and Nicole Cook, PhD, MPA[2]

Appendix Figure 1

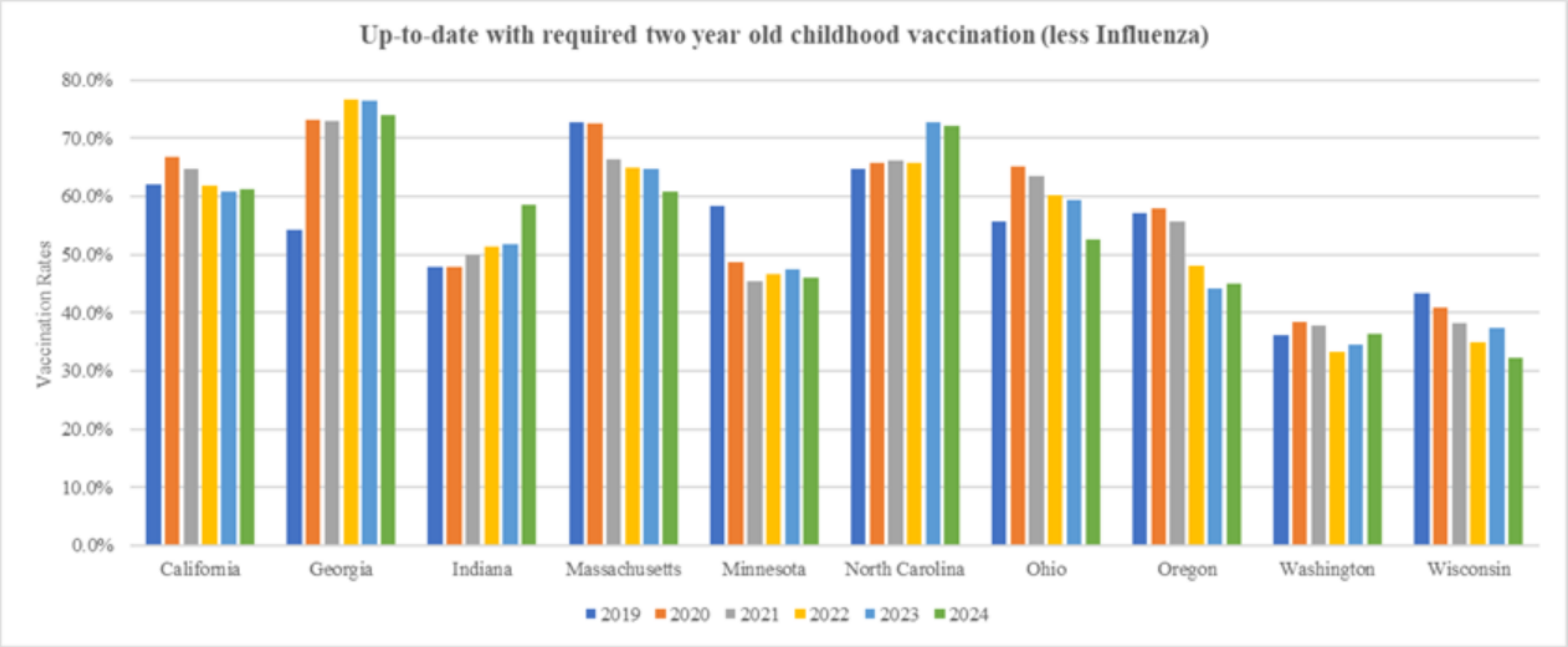

Appendix Figure 2

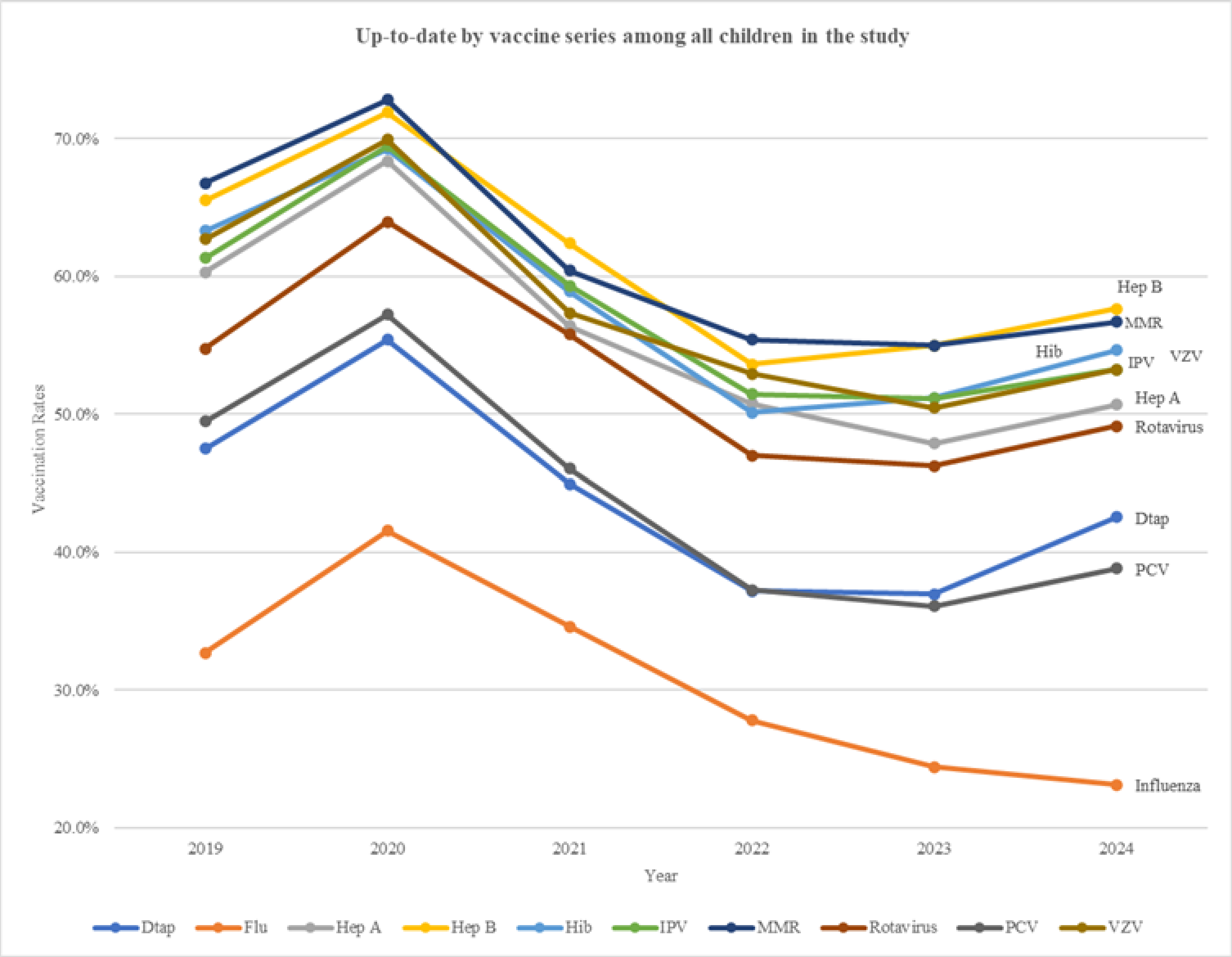

Appendix Figure 3

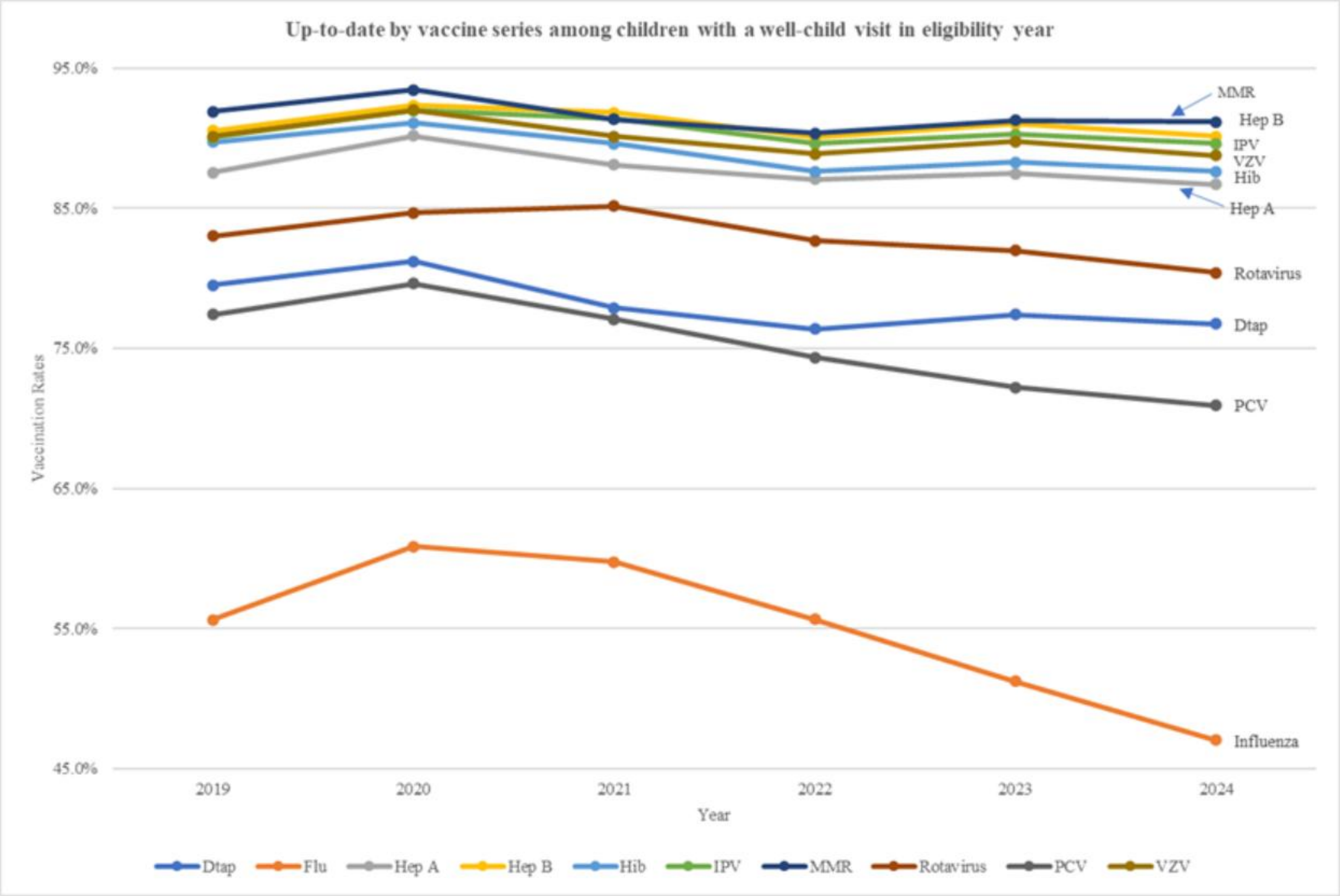

Supplement: Supplementary file 1 [file mmc1.pdf]
